# Practical Notes and Formulæ

**Published:** 1880-11-20

**Authors:** 


### PRACTICAL NOTES AND FORMULÆ.

**Compound Syrup of Hypophosphites.**—Among the various methods for making this product, we have revamped a little by giving a cut here and placing an addition there, and arranged a formula that works very satisfactorily, which is as follows:

| | |
|---|---|
| Sulphate of iron C. P. | 6 dr. |
| Hypophosphite of lime | 3½ dr. |
| Boiling distilled water | 18 oz. |

The sulphate of iron is dissolved in about 1 oz. of the water and filtered, and the hypophosphite in the balance of the water and filtered; the two solutions are mixed in a pint bottle and allowed to stand for one hour until all the hypophosphite of iron is formed; at the end of the stated time the magma is turned upon a filter of linen, pressed and filtered afresh through paper, and sufficient distilled water is added to make the measure 18 oz. In this way of introducing the hypophosphite of iron into the syrup, we have our iron freshly made, and at the same time in solution in readiness to combine directly with the other salts, which are as follows:

| | |
|---|---|
| Hypophosphite of Calcium | 768 grs. |
| " " Sodium | 512 " |
| " " Potassium | 256 " |
| Sugar | 22 oz. |

The solution of the hypophosphite of iron is first heated to the boiling point, and in it are dissolved the salts of soda, lime and potash, by the aid of two drachms of citric acid,* which helps to dissolve all of the lime salt. The solution is thus filtered, and in it is dissolved the given amount of sugar. Each teaspoonful contains:

3 grains of hypophosphite of lime.
2 " " " " soda.
1 " " " " potash.
2 " " " " iron.

The citric acid is used for the purpose of dissolving the calcium salt, also of making the iron taste, giving to the finished syrup an agreeable tartness.—*Proc. of the Cal. Phar. Assn.*

*Hypophosphorous acid is both chemically and therapeutically preferable.—ED.

**Nitrite of Amyl in Uterine Hemorrhage.**—Dr. Kerr reports a case of severe post-partum uterine hemorrhage, in which the patient was restored from a state of collapse by the inhalation of five minims of nitrite of amyl, whilst the hemorrhage was immediately arrested. The author was led to adopt this method of treatment by the report of Dr. Roehler's use of warm fomentations to the head in cases of uterine hemorrhage, to prevent anæmia of the brain and of the heart. In either case the *rationale* of the treatment is probably to be found in the rapid dilatation of the cerebral vessels.—*Brit. Med. Jour.*

**Cuticura Resolvent.**—Each fluid ounce weighs 453.6 grains, and leaves, on evaporation, a residue weighing 25.17 grains, equivalent to 5.55 per cent. This residue consists of:

| | | |
|---|---|---|
| Organic matter | 21.86 | grains. |
| Potassic iodide | 2.13 | " |
| Soluble salts, not iodide | 64 | " |
| Insoluble ash (in water) | 54 | " |
| | 25.17 | grains. |

The organic matter gave distinctive tests for aloin, chrysophanic and iron-greening tannic acid, which reactions, taken together with taste, odor and physical properties, point to the presence of aloes and rhubarb.

The following prescription would closely imitate the "Resolvent:"

Take of:

Socotrine aloes,
Rhubarb, each........................................ 1 drachm.
Potassic iodide........................................36 grains.
Whiskey........................................ 1 pint.

Macerate over night and filter.—*New Remedies, June*, 1880.

**How to Make a Poultice.**—The common practice in making poultices of mixing the linseed meal with hot water and applying it directly to the skin is quite wrong; because, if we do not wish to burn the patient, we must wait until a great portion of the heat has been lost. The proper method is to take a flannel bag of the size of the poultice required, to fill this with the linseed poultice as hot as it can possibly be made, and to put between this and the skin a second piece of flannel, so that there shall be at least two thicknesses of flannel between the skin and the poultice itself. Above the poultice should be placed more flannel, or a piece of cotton wool, to prevent it from getting cold. By this method we are able to apply the linseed meal boiling hot without burning the patient, and the heat, gradually diffusing through the flannel, affords a grateful sense of relief, which cannot be obtained by other means. There are few ways in which such marked relief is given to abdominal pain as by the application of a poultice in this manner.—DR. BRUNTON, *in Brain.*

**Odorless Iodoform.**—No satisfactory method of disguising the penetrating odor of iodoform has yet been proposed. The following formula, however, it is asserted by Dr. Lindemann, (Allgemeine Medicin Central-Zeitung, 1879, No. 74), does away with the smell entirely.

℞ Iodoform........................................grs. xvj.
Bals. peruviani........................................grs. xxx.
Vaselini, adipis, ung. glicerini, aa........................................℥ ij.

If desired in a fluid form, the following formula may be employed:

℞ Iodoform........................................grs. xvj.
Bals. peruviani........................................℈ ijss.
Alcoholis, glycerini, collodii, aa........................................℥ iij.

**Soothing Ointment.**—The following we find in the London Specialist:

By far the best of all the soothing ointments with which I am acquainted is composed of:

℞ Bismuthi oxidi.......................................... ʒ j

Acidi oleici.......................................... ʒ xiij.

Cerae albae.......................................... ʒ iij.

Vaselini.......................................... ʒ ix.

Olei rosae.......................................... m v.

I have not only used this ointment with the very best results myself, but those of my professional brethren to whom I have recommended it have professed themselves equally satisfied with it; and one medical man in particular recently informed me that it was the only ointment, of the many which he had tried, which had proved a sedative in his own case.

Instead of merely rubbing soothing ointments upon the inflamed surfaee, as is so often done, it is always preferable, when at all possible, to apply them spread thickly upon a piece of linen, which should not be too large, else they do not lie evenly upon the inflamed parts.

**Erysipelas.**—Dr. Rothe, in Memorabilien, says: I have been accustomed, for years, to employ painting of the inflamed surface and its surrounding parts, every two hours, with a mixture of carbolic acid and oil of turpentine:

℞ Acidi carbolici.......................................... 1 part.
Spiritus vini.......................................... 1 "
Olei terebinthinae.......................................... 2 "
Tincture iodini.......................................... 1 "
Glycerine.......................................... 5 "

and have had occasion to be well satisfied with its effects.

The applications are entirely painless, and do not even excite heat of the skin. Commonly this is found wrinkled and pale on the second day. This method does not check the advance of the redness and swelling any more surely than any other; but a part newly attacked can be restored equally as quickly to its normal condition by the same application, so that the course of an ordinary facial erysipelas is usually terminated in three or four days.

The part that has been painted is covered with a very thin layer of fine carded cotton batting. In case of high fever, or gastritis, of course, the remedies indicated—digitalis, quinine, an emetic, etc.,—must be employed.

**Abrasions of the Cornea.**—Dr. Dyer, in Medical Herald, says: Abrasions of the cornea, the result of injury, require a simple anodyne treatment:

℞ Atropiae sulphatis.......................................... gr. ss. ij.
Morphiae sulphatis.......................................... gr. ij. iv.
Aquae dest.......................................... ℥ i,

M. Fiat solutio,

**Arsenic in Neuralgia.**--Dr. J. T. Stewart, in Peoria Medical Monthly, says: "One old and tried remedy is worth a thousand of the new ones that are thrust upon the profession with the sound of trumpets by manufacturers of drugs all over the country. This statement is pre-eminently true as to arsenic in ordinary neuralgia. To derive the full benefit of this drug it must be given, at first, in small and frequently-repeated doses, gradually increasing the dose, until the neuralgia is controlled or the medicine begins to show its constitutional effects.

"Another point of equal importance is this: Long experience has taught me that by combining it with opium a much larger quantity will be tolerated by the system than would be if given alone. The opium aids it also in controlling the neuralgia. The following is a formula which I have used with great satisfaction for many years:

| | | |
|---|---|---|
| ℞ | Fowler's solution | 3 drachms. |
| | Tinct. opii | 1½ " |
| | Alcohol | 1⅓ " |

Mix.

"The alcohol, of course, is added to prevent so much of the opium in the laudanum from being precipitated. In giving this mixture I am in the habit of beginning with ten drops in water every three hours, and increasing two or three drops a day until the disease is controlled, or until decided constitutional effects of arsenic are produced. It is rarely that the neuralgia does not yield before the constitutional effects of the drug are manifest.—*Indiana. Med. Rep.*

**Chloral in the Vomiting of Pregnancy.**—Dr. Hertsberg, of the Berlin Charite, in Berlin Klin. Woch., calls attention to the great efficacy of chloral in vomiting in the early months of pregnancy. He always uses the following formula:

| | | |
|---|---|---|
| ℞ | Chloral hydratis | gr. xviij |
| | Syr. aurant. cort | ʒ iv |
| | Aquæ | ℥ iiss |

Dose, Tablespoonful every two hours until vomiting stops.—*Med. and Surg. Reporter.*

**Wound-Dressing.**—Samton Gamgee, F.R.C.S., in the Lancet, says: "I do not dispute, as for many generations has been admitted, that antiseptics are of service in surgical practice; but they are accessories, not essentials. The essentials for successful wound-treatment are *accurate coaptation, dry and infrequent dressing, uniform, gentle pressure, and absolute rest.*—*Canadian Jour.*

**Venereal Warts.**—Equal parts of burnt alum and tannin sprinkled in powder upon venereal warts will desiccate them, and they can be rubbed off in a few days.—*Canada Med. Rec.*, Sept., 1880.

**Good Waterproof Varnish** may be made by dissolving 1 oz. of india-rubber to a jelly in one pint of turpentine; then add one pint of hot linseed oil and mix over a slow fire.

